# Identification of potential biomarkers and pathways for asthenozoospermia by bioinformatics analysis and experiments

**DOI:** 10.3389/fendo.2024.1373774

**Published:** 2024-05-28

**Authors:** Hui Lu, Liqiang Zhao, Anguo Wang, Hailing Ruan, Xiaoyan Chen, Yejuan Li, Jiajia Hu, Weiying Lu, Meifang Xiao

**Affiliations:** ^1^ Reproductive Medicine Center, Hainan Women and Children’s Medical Center, Haikou, Hainan, China; ^2^ Department of Clinical Laboratory, Center for Laboratory Medicine, Hainan Women and Children’s Medical Center, Haikou, Hainan, China

**Keywords:** asthenozoospermia, WGCNA, GSEA, autophagy, M6A

## Abstract

**Background:**

Asthenozoospermia, a type of male infertility, is primarily caused by dysfunctional sperm mitochondria. Despite previous bioinformatics analysis identifying potential key lncRNAs, miRNAs, hub genes, and pathways associated with asthenospermia, there is still a need to explore additional molecular mechanisms and potential biomarkers for this condition.

**Methods:**

We integrated data from Gene Expression Omnibus (GEO) (GSE22331, GSE34514, and GSE160749) and performed bioinformatics analysis to identify differentially expressed genes (DEGs) between normozoospermia and asthenozoospermia. Gene Ontology (GO), Kyoto Encyclopedia of Genes and Genomes (KEGG) pathway analyses were conducted to gain insights into biological processes and signaling pathways. Weighted Gene Co-expression Network Analysis (WGCNA) identified gene modules associated with asthenozoospermia. Expression levels of key genes were assessed using datasets and experimental data. Gene Set Enrichment Analysis (GSEA) and correlation analysis identified pathways associated with the hub gene and explore the relationship between the *ZNF764* and *COQ9* and mitochondrial autophagy-related genes. Competitive endogenous RNA (ceRNA) networks were constructed, and *in vitro* experiments using exosome samples were conducted to validate this finding.

**Results:**

*COQ9* was identified as a marker gene in asthenozoospermia, involved in autophagy, ATP-dependent chromatin remodeling, endocytosis, and cell cycle, etc. The ceRNA regulatory network (LINC00893/miR-125a-5p/COQ9) was constructed, and PCR demonstrated that *LINC00893* and *COQ9* were downregulated in asthenozoospermia, while miR-125a-5p and m6A methylation level of *LINC00893* were upregulated in asthenozoospermia compared to normozoospermic individuals.

**Conclusion:**

The ceRNA regulatory network (*LINC00893*/miR-125a-5p/*COQ9*) likely plays a crucial role in the mechanism of asthenozoospermia. However, further functional experiments are needed to fully understand its significance.

## Introduction

Infertility is a prevalent issue affecting a significant proportion of the global population, with male infertility accounting for approximately 40–50% of all infertility cases (1). Among the various causes of male infertility, asthenozoospermia stands out as a common condition characterized by reduced sperm motility, primarily attributed to dysfunctional sperm mitochondria (2, 3). Extensive research has highlighted the involvement of genetic factors (4), environmental factors such as infections (e.g., human papillomavirus (HPV) infection (5) and novel coronavirus disease (COVID-19) infection (6)), varicocele (7), and lifestyle (8) in the development of asthenozoospermia. However, the precise molecular mechanisms underlying this condition remain poorly understood. Therefore, gaining insights into the underlying molecular mechanisms and identifying potential biomarkers for asthenozoospermia are crucial for the development of effective diagnostic and therapeutic strategies.

Recently, exosomes have garnered significant attention as a crucial medium for intercellular communication (9). These small extracellular vesicles, ranging from 30 to 150 nanometers in diameter, are released by cells and carry a diverse cargo of bioactive molecules, including microRNAs (miRNAs), long noncoding RNAs (lncRNAs), and messenger RNA (mRNA) (10). Exosomes have been shown to facilitate information exchange between cells and participate in various physiological and pathological processes by delivering these molecules (11). In the male reproductive tract, exosomes are secreted and are believed to play a crucial role in sperm maturation and function. Notably, ejaculated sperm have the ability to capture exosomes. Studies have demonstrated that exosomes derived from normozoospermic individuals can enhance sperm motility and promote capacitation, while exosomes from males with severe asthenozoospermia lack similar effects (12). Our previous research has provided initial insights into the dysregulated competitive endogenous RNA (ceRNA) network in asthenozoospermia, highlighting the potential involvement of exosomal lncRNAs in the pathogenesis of this condition (13). Although previous bioinformatics analyses have identified potential key lncRNAs, miRNAs, hub genes, and crucial pathways associated with asthenozoospermia (14–16), there are still many unexplored molecular mechanisms and potential biomarkers that related to asthenozoospermia need to be elucidated.

**Figure 1:**
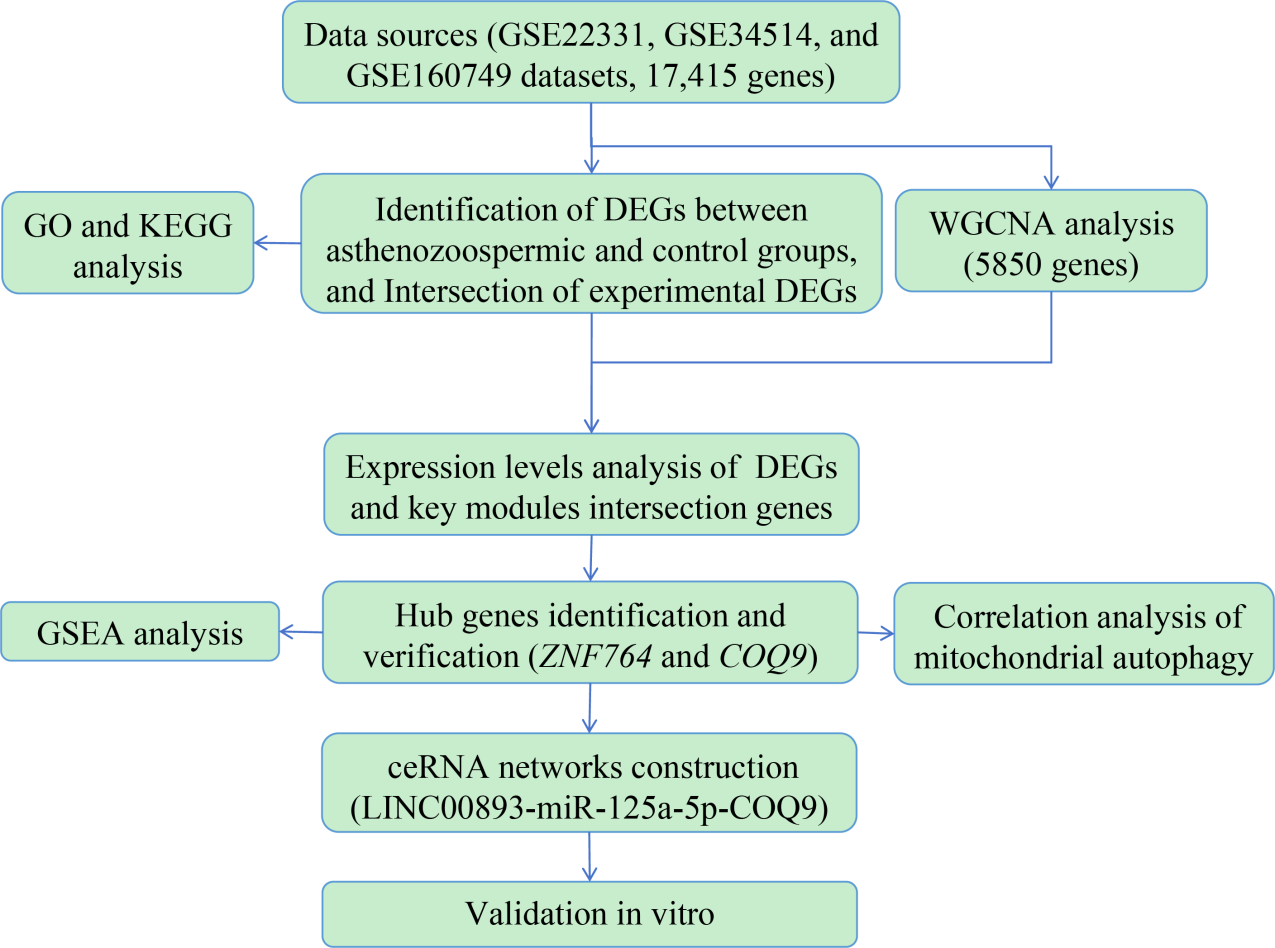
The design and analysis flowchart to identify potential biomarkers and biological pathways in asthenozoospermia. DEGs, differentially expressed genes; GO, Gene Ontology; KEGG, Kyoto Encyclopedia of Genes and Genomes; WGCNA, Weighted Gene Co-expression Network Analysis; GSEA, Gene Set Enrichment Analysis; ceRNA, Competitive endogenous RNA.

Therefore, the aim of this study was to identify potential biomarkers and pathways associated with asthenozoospermia through a combination of bioinformatics analysis and experimental validation *in vitro*. To achieve this, we integrated data from multiple sources, including GSE22331, GSE34514, and GSE160749, and identify the differentially expressed genes (DEGs) between normozoospermic and asthenozoospermic groups. To gain insights into the biological processes and signaling pathways related to the intersection genes between bioinformatics DEGs and previous experimental DEGs, we performed Gene Ontology (GO) and Kyoto Encyclopedia of Genes and Genomes (KEGG) pathway analyses. Additionally, we utilized Weighted Gene Co-expression Network Analysis (WGCNA) to identify gene modules closely associated with asthenozoospermia. We assessed the expression levels of the intersection genes between DEGs and WGCNA genes using the datasets and our previous experimental data. Gene Set Enrichment Analysis (GSEA) was conducted to identify pathways associated with the hub gene. Furthermore, we explored the relationship between the hub gene and mitochondrial autophagy, a process known to play a crucial role in sperm motility (17), through correlation analysis. Competitive endogenous RNA (ceRNA) networks were constructed to uncover the regulatory relationships among these molecules. To validate the findings from our bioinformatics analysis, we performed *in vitro* experiments using exosome samples. The design and analysis flowchart of our study is shown in **Figure 1**. These comprehensive methods aim to identify potential biomarkers and pathways associated with asthenozoospermia, providing a deeper understanding of the molecular mechanisms underlying asthenozoospermia.

## Materials and methods

### Data collection and preprocessing

We searched gene expression profiling datasets for asthenozoospermia using the term “asthenozoospermia” from the Gene Expression Omnibus (GEO) publicly available database (https://www.ncbi.nlm.nih.gov/gds/). The inclusion criteria for the dataset are as follows: (1) the dataset includes whole genome-wide expression mRNA microarray data; (2) the samples were sperm from normozoospermic and asthenozoospermic; (3) the organism was restricted to Homo sapiens. Finally, multiple gene expression datasets, including GSE22331 (1 normozoospermia and 1 asthenozoospermia), GSE34514 (4 normozoospermias and 4 asthenozoospermias), and GSE160749 (6 normozoospermias and 5 asthenozoospermias), were downloaded and integrated for further analysis, which included a total of 11 normozoospermias and 10 asthenozoospermias (**Supplementary Table 1**). Firstly, we conducted independent data cleaning and preprocessing on each dataset, including outlier detection, missing value filling, etc., to ensure data consistency and integrity. Additionally, the removeBatchEffect function of the R software (version 4.2.1) “limma” package was utilized for batch correction to minimize the batch effect. By reducing the dimensionality of the data, Principal Component Analysis (PCA) allowed us to visualize the overall patterns and relationships within the dataset (**Supplementary Figure S1**). This analysis helped confirm that the integrated data remained reliable and informative, despite the potential confounding effects of batch variations.

### Identification of key DEGs

DEGs were identified using the “limma” packages of the R language, with a significance threshold of *p*-value < 0.05 and |log2FC| > 0.5. Volcano plots were generated using the SangerBox online software (http://sangerbox.com/home.html) based on the “ggplot” package of R language. Venn diagrams (https://jvenn.toulouse.inrae.fr/app/example.html) were used to identify the key DEGs by intersecting the DEGs obtained from GEO databases with our previous experimental DEGs (1,128 up-regulated and 1,210 down-regulated) (13).

### GO and KEGG enrichment analysis

To explore the biological functions and pathways associated with the key DEGs, we conducted GO and KEGG analyses using the “AnnotationDbi”, “clusterProfiler”, and “ggplot2” R packages. A significance threshold of *p*.adjust < 0.05 was applied to determine significant terms. The results were visualized to provide insights into the enriched biological processes and pathways associated with the DEGs.

### Co-expression network construction

A total of 5850 genes with a variance greater than 0.5 in the integrated dataset (11 normozoospermic and 10 asthenozoospermic individuals) were selected for WGCNA. First, we calculated the similarity between samples using horizontal hierarchical clustering. Then, we constructed an adjacency matrix and determined the optimal soft threshold power (β = 14) based on scale independence and mean connectivity (scale free R^2 =^ 0.80). Next, we transformed the adjacency matrix into a topological overlap matrix (TOM) to assess network interconnectedness. The dynamic tree cutting method was applied to identify co-expressed gene modules, and a cluster dendrogram was generated to visualize the modules represented by different colors using hierarchical clustering. To ensure reliable results, we set the minimum number of genes per module to 50 and the module cutting height to 0.1. Pearson correlation analysis was performed to calculate the correlations between module eigengenes (MEs) and phenotypes, identifying the module with the highest positive correlation with asthenozoospermia. The genes most associated with the darkolivegreen and skyblue modules and asthenozoospermic traits were selected for further analysis. Additionally, gene significance (GS) and module membership (MM) values were generated, and genes with |MM| > 0.8 and |GS| > 0.4 were considered hub genes in the key module.

### Identification and expression hub genes

The gene that exhibited the most significant difference, which was found at the intersection of DEGs and the hub genes identified by WGCNA, was selected as the core gene for further investigation. To visualize the expression patterns of the intersecting genes between DEGs and the hub genes identified by WGCNA, we generated violin plots using SangerBox software (http://sangerbox.com/home.html) based on datasets and our previous experimental data. These plots provided a graphical representation of the expression levels of the selected genes, allowing for a better understanding of their differential expression in asthenozoospermic and normozoospermic groups.

### Single-gene GSEA of the hub gene

To gain further insights into the functional implications of hub gene, we performed single-gene GSEA and visualization using the R packages “clusterProfiler”, “enrichplot”, and “stringr”. Firstly, we divided all samples into low-expression and high-expression groups based on the expression levels of the key gene. Next, we conducted GSEA to identify significantly different KEGG pathways between the two groups. Pearson correlation analysis was performed to assess the correlation between the hub gene and all genes in the integrated dataset, and the genes were ranked in descending order based on the correlation scores. The ranked genes were then used as the gene set to be tested, while the KEGG signaling pathway served as the predefined gene set. Finally, we examined the enrichment of the KEGG signaling pathway in the corresponding set of genes to be tested, considering a *p*.adjust value less than 0.05 as the cutoff criterion for statistical significance. This analysis provided valuable information about the potential functional associations and pathways related to hub gene.

### Association with autophagy

To explore the potential association between the hub gene and mitochondrial autophagy, we obtained a set of 29 mitochondrial autophagy-related genes (including *ATG12*, *FUNDC1*, *SQSTM1*, *TOMM5*, *UBB*, *ATG5*, *MAP1LC3A*, *PGAM5*, *SRC*, *UBC*, *MAP1LC3B*, *PINK1*, *TOMM20*, *ULK1*, *CSNK2A2*, *MFN1*, *PARK2*, *TOMM22*, *TOMM70A*, *VDAC1*, *CSNK2B*, *MFN2*, *RPS27A*, *TOMM40*, and *UBA52*) from the Reactome Pathway Database (https://reactome.org). We then performed Pearson correlation analysis to assess the correlation between the key gene and these mitochondrial autophagy-related genes. The resulting correlation heatmap was visualized using the SangerBox software. *p* less than 0.05 was considered as cut-off criterion for statistically significant.

### ceRNA regulatory network construction

To investigate the potential regulatory interactions between the key gene and non-coding RNAs, we utilized online databases such as miRcode (http://www.mircode.org/), TargetScan (https://www.targetscan.org/vert_80/), and starBase (https://starbase.sysu.edu.cn/), along with our previous research findings. These resources allowed us to predict miRNAs and lncRNAs that may be associated with the key gene. We then constructed lncRNA-miRNA-mRNA networks by integrating the lncRNA-miRNA and mRNA-miRNA interactions. The resulting networks were visualized using Cytoscape software (version 3.10.0), providing a comprehensive view of the potential regulatory relationships between the key gene and non-coding RNAs.

### Extraction and identification of seminal plasma exosomes

We collected semen samples from 3 asthenozoospermia males and 3 normozoospermia males at the Hainan Women and Children’s Medical Center. The collection methods, diagnosis, and inclusion criteria followed the protocols outlined in our previous study (13). To isolate seminal plasma exosomes, the semen samples were rapidly thawed at 37°C and transferred to a new centrifuge tube. They were then centrifuged at 4°C, 2000 ×g for 30mins. The supernatant was carefully transferred to another tube and subjected to a second centrifugation at 4°C, 10,000 ×g for 45mins to remove larger vesicles. The resulting supernatant was filtered using a 0.45μm filter membrane, and the filtrate was collected. Subsequently, the filtrate was transferred to a new centrifuge tube and centrifuged at 4°C, 100,000 ×g for 70mins. After removing the supernatant, the pellet was resuspended in 10 mL of pre-cooled 1× PBS and subjected to another centrifugation at 4°C, 100,000 ×g for 70mins. Finally, the supernatant was discarded, and the exosome pellet was resuspended in 100μL of pre-cooled 1× PBS and stored at -80°C. To examine the structural characteristics and morphology of the isolated exosomes, we performed high-resolution imaging using transmission electron microscopy (TEM, HT-7700, Hitachi) (**Supplementary Figure S2A**). Additionally, the size distribution and concentration of the exosomes were determined using a NanoFCM N30E particle size analyzer (**Supplementary Figures S2B, C**).

### Quantitative reverse-transcription polymerase chain reaction

Total RNA was extracted from exosomes of asthenozoospermia and normozoospermia using TRIZOL reagent (Invitrogen, Carlsbad, CA, USA) following the manufacturer’s instructions. The concentration and purity of RNA were assessed using a NanoDrop 2000 spectrophotometer (Thermo Fisher Scientific, Waltham, MA, USA) by measuring the optical density (OD) at 280 and 260 nm. The integrity of the RNA was evaluated using agarose gel electrophoresis. Subsequently, the RNA was reverse transcribed into complementary DNA (cDNA) using a reverse transcription kit (Nocoprotein Co., Ltd., Suzhou, China). For qRT-PCR, the SYBR Green Master Mix (Applied Biosystems, MA, USA) and the Applied Biosystems 7500 sequence detection system were used. The cycling conditions consisted of an initial denaturation step at 95°C for 1 minute, followed by 45 cycles of denaturation at 95°C for 20 seconds, and annealing/extension at 60°C for 45 seconds. U6 was employed as an endogenous control for miRNA, while GAPDH served as the internal control for mRNA and lncRNA. The relative gene expression levels were calculated using the 2^−ΔΔCt^ method. The primer sequences used for qRT-PCR are provided in **Table 1**.

**Table 1:** The primer sequences used for qRT-PCR.

Primer names	Sequences
ZNF764-F	GCCAAACACCAGTGGGTTCATC
ZNF764-R	GGGTCAGGGTCACAGACAGAC
COQ9-F	ACTTGGCGCTTCCTGGAAAACC
COQ9-R	ACCCATGAGTCCTTGCACCAGT
LINC00893-F	CAGATCTCCATGCAAAGTATGTC
LINC00893-R	GTTAGAATTATCTTCAAGGAGCCTC
GAPDH-F	GGAGCGAGATCCCTCCAAAAT
GAPDH-R	GGCTGTTGTCATACTTCTCATGG
miR-125a-5p	TCCCTGAGACCCTTTAACCTGTGA
U6-F	GGAACGATACAGAGAAGATTAGC

F, Forward primer; R, Reversed primer.

### Relative quantitative detection of m6A methylation of lncRNA

The relative quantitative detection of m6A methylation involved the following steps: 1) Total RNA extraction and quality control: Total RNA was extracted as previously described, and quality control measures were performed to ensure RNA integrity. 2) RNA purification and fragmentation: The extracted total RNA was treated with DNase I to remove any trace genomic DNA (gDNA). Exogenous positive and negative m6A-modified fragments were introduced as controls. The RNA was then fragmented into fragments ranging from 60 to 200 base pairs (bp) by fragmenting reagents in the presence of metal ions. 3) Antibody immunoprecipitation: Dynabeads Protein A was mixed with the anti-m6A antibody to form complexes. The fragmented RNA was incubated with the bead-antibody complex, allowing for the binding of m6A-modified RNA fragments. The beads were separated from the solution using a magnet, and then washed to remove non-specifically bound RNA fragments. The m6A-modified RNA fragments were extracted using a lysis buffer. 4) Reverse transcription of RNA: A reverse transcription reaction was performed using random primers to synthesize single-stranded cDNA. This cDNA served as a quantitative template for subsequent analysis. 5) qRT-PCR: qRT-PCR was carried out using m6A-modification site-specific primers. SYBR fluorescent dye was used for detection, and ROX dye was added for inter-well signal correction. The reaction was amplified on a Real-Time PCR instrument, and the amplification and melt curves were analyzed. The primer sequences are provided in **Table 2**.

**Table 2:** The primer sequences used for m6A methylation detection.

Primer types	Primer names	Sequences	Covering m6A site
Verify primers	LINC00893_F	AATTCGTCCTGCTTCCCTGG	658
	LINC00893_R	CACCTTATCACTGGCTGCCA	
Exogenous negative sites	Cluc_F	AAGCAACTGCTCGTCGTACA	/
	Cluc_R	ACCGCAAATACCGCAAGTCT	
Exogenous positive sites	Gluc_F	CTGTCTGATCTGCCTGTCCC	/
	Gluc_R	TTGTCGCCTTCGTAGGTGTG	
Endogenous positive sites	SLC39A14-Human_F	CCAGATTGGGTAGGGCTCTG	/
	SLC39A14-Human_R	GATGGTAAGTCCTCGGGCTG	

F, Forward primer; R, Reversed primer.

## Results

### Identification of key DEGs

**Figure 2:**
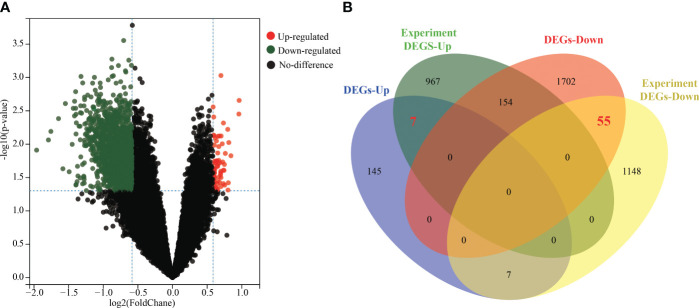
Identification of key DEGs between asthenozoospermic and normozoospermic groups. **(A)** Volcano plots of DEGs. The x-axis represents the log2 fold change (log2FC) value, and the y-axis represents significant difference. The red dots represent significantly upregulated genes, the green dots represent significantly downregulated genes, the black dots represent no significantly. **(B)** The common key genes screened from DEGs of datasets and previous experiment are shown in Venn diagram. DEGs, differentially expressed genes.

Normalization and integration of the mRNA expression datasets GSE22331, GSE34514, and GSE160749 resulted in a total of 17,415 genes. A total of 2,070 DEGs were identified between asthenozoospermic and normozoospermic groups, including 159 upregulated genes and 1,911 downregulated genes. The volcano plot visualized these results, with each dot representing a gene (**Figure 2A**). Additionally, key DEGs (containing 7 upregulated genes and 55 downregulated genes) were identified by finding the intersection between the DEGs from the datasets and previous experiments (**Figure 2B**).

### GO and KEGG analyses of the key DEGs

**Figure 3:**
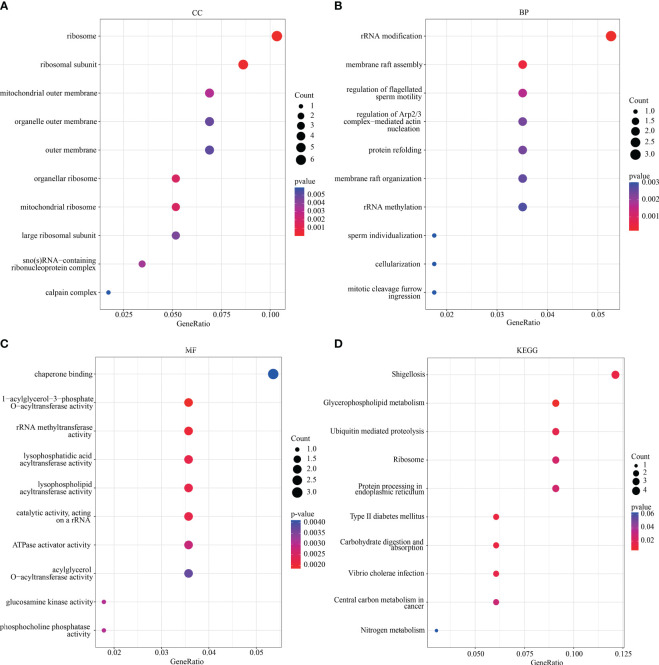
GO and KEGG analysis. The visualization results of **(A–C)** partial GO biological function analysis and **(D)** partial KEGG analysis of magenta module gene. The first 10 important enrichment pathways are shown. GO, Gene Ontology; KEGG, Kyoto Encyclopedia of Genes and Genomes.

GO and KEGG analyses were performed to determine the potential biological functions and pathways associated with the key DEGs (**Figure 3**). The GO analysis revealed that most of the DEGs were located in the ribosome (organellar, mitochondrial), ribosomal subunit, outer membrane (mitochondrial and organelle), and calpain complex (**Figure 3A**), which were involved in processes such as rRNA modification and methylation, membrane raft assembly, regulation of flagellated sperm motility, sperm individualization, cellularization (**Figure 3B**). Furthermore, many DEGs were found to be involved in the regulation of enzyme activity, including rRNA methyltransferase, lysophosphatidic acid acyltransferase, and glucosamine kinase (**Figure 3C**). The KEGG analysis showed that the key DEGs were mainly associated with glycerophospholipid metabolism, ubiquitin-mediated proteolysis, and protein processing in the endoplasmic reticulum (**Figure 3D**).

### Co-expression network construction

**Figure 4:**
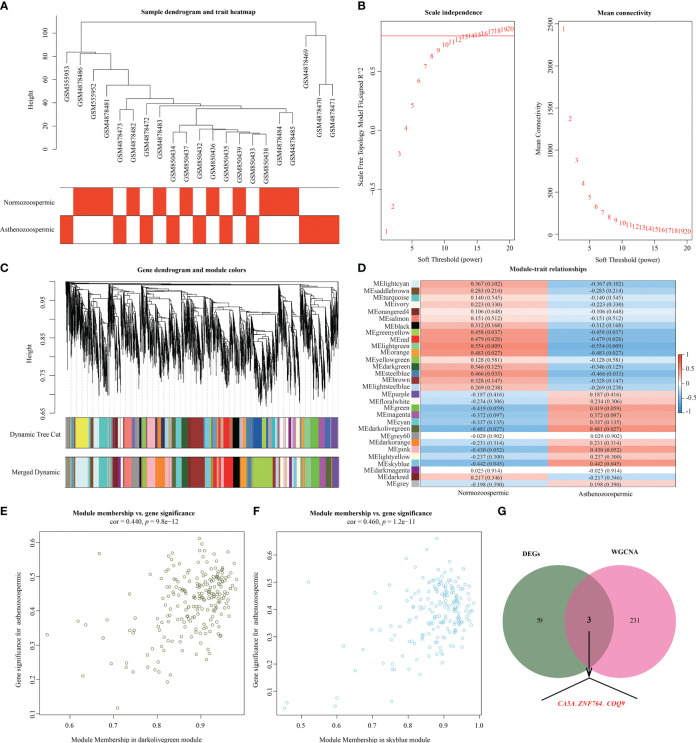
Weighted gene co-expression network analysis. **(A)** Clustering dendrogram of 21 samples. **(B)** Analysis of the scale-free index (left) and mean connectivity (right) for various soft-threshold powers (β). **(C)** Clustering dendrogram. Each branch represents one gene, and each color at the bottom represents one coexpression module. **(D)** Heatmap of the correlation between module eigengenes and clinical phenotype. The upper number in the color grid represents the correlation as well as its *p*-value. **(E)** The scatterplot of module membership and gene significance in darkolivegreen module. **(F)** The scatterplot of module membership and gene significance in skyblue module. **(G)** Venn diagram for key genes from DEGs and key modules. DEGs, differentially expressed genes.

A total of 5,850 genes with larger variance were selected for co-expression analysis based on the variance results of gene expression. Using the Pearson’s correlation coefficient, all samples from GSE22331, GSE34514, and GSE160749 were clustered, resulting in a sample clustering tree (**Figure 4A**). With a threshold correlation coefficient of 0.80 and a soft threshold of 14, a scale-free topology module was constructed (**Figure 4B**). Based on average hierarchical clustering and dynamic tree clipping, thirty co-expression modules were created (**Figure 4C**). Correlation analysis between the modules and clinical traits revealed that the darkolivegreen (r = 0.481, *p* = 0.027) and skyblue (r = 0.442, *p* =  0.045) modules showed modest significant correlations with asthenozoospermia (**Figure 4D**). GS and MM analysis identified the darkolivegreen (correlation coefficient = 0.440, *p*  = 9.8 × 10^−12^; **Figure 4E**) and skyblue (correlation coefficient = 0.460, *p*  = 1.2 × 10^−11^; **Figure 4F**) modules as important modules strongly correlated with asthenozoospermia for further analysis. According to the cutoff criteria (|MM|> 0.8 and |GS|> 0.4), the skyblue module contained 196 genes, of which 93 genes were identified as hub genes, and 141 hub genes were identified in the darkolivegreen module (218 genes) (**Supplementary Table 2**). A Venn diagram was then developed to take the intersection between the above genes and key DEGs to identify hub genes in asthenozoospermia, which were *ZNF764*, *COQ9* and *CA5A*, respectively (**Figure 4G**).

### Verification of key genes expression and GESA

**Figure 5:**
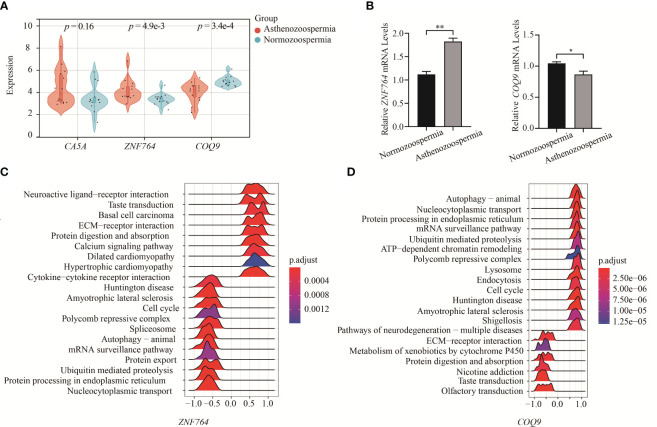
Verification of key genes expression and GESA. **(A)** Verification of key genes expression between asthenozoospermic and normozoospermic groups. **(B)** The mRNA expression levels of *ZNF764* and *COQ9* in seminal exosomes of asthenozoospermic and normozoospermic groups via qRT-PCR. **(C)** Ridge map of *ZNF764* most related pathways via single gene GSEA. **(D)** Ridge map of *COQ9* most related pathways via single gene GSEA. GSEA, Gene Set Enrichment Analysis. *p < 0.05, **p < 0.01.

The expression levels of hub genes in asthenozoospermia and normozoospermia were validated using the integrated GO datasets (GSE22331, GSE34514, and GSE160749) and previous experimental data (**Figure 5A**). The expression level of *ZNF764* was found to be higher in asthenozoospermic patients compared to normozoospermic individuals (*p* = 4.9e-3), while the expression of *COQ9* was significantly decreased in asthenozoospermic patients (*p* = 3.4e-4). However, no significant differences were observed in the expression level of *CA5A* between the two groups (*p* = 0.16). Furthermore, the expression of these two genes (*ZNF764* and *COQ9*) in seminal exosomes of asthenozoospermic and normozoospermic individuals was validated through RT-PCR (**Figure 5B**).

Furthermore, we performed GSEA to gain a more accurate understanding of the functional roles of the hub genes. Based on the designated cut-off criteria, we identified the top twenty KEGG pathways enriched in the analysis. For *ZNF764*, the enriched pathways included nucleocytoplasmic transport, protein processing in endoplasmic reticulum, ubiquitin mediated proteolysis, protein export, mRNA surveillance pathway, autophagy-animal, spliceosome, polycomb repressive complex, and cell cycle, etc. (**Figure 5C**). For *COQ9*, the enriched pathways included autophagy, nucleocytoplasmic transport, protein processing in endoplasmic reticulum, mRNA surveillance pathway, ubiquitin mediated proteolysis, ATP-dependent chromatin remodeling, polycomb repressive complex, lysosome, endocytosis, and cell cycle, etc. (**Figure 5D**).

### Association with autophagy related genes

We analyzed the correlation between expression levels of *ZNF764* and *COQ9* and the expression levels of 22 mitochondrial autophagy-related genes (**Table 3**; **Supplementary Figure S2**). Significant negative correlations were found between the expression level of *ZNF764* and three mitochondrial autophagy-related genes, including *CSNK2A2* (cor = -0.792, *p* = 0.006), *MFN1* (cor = -0.789, *p* = 0.007), and *PARK2* (cor = -0.775, *p* = 0.009). On the other hand, the expression level of *COQ9* showed significant positive correlations with 16 mitochondrial autophagy-related genes, including *SQSTM1* (cor = 0.955, *p* < 0.001), *TOMM5* (cor = 0.903, *p* < 0.001), *UBB* (cor = 0.772, *p* = 0.009), *ATG5* (cor = 0.744, *p* = 0.014), *MAP1LC3A* (cor = 0.914, *p* < 0.001), *PGAM5* (cor = 0.810, *p* = 0.005), *UBC* (cor = 0.804, *p* = 0.005), *MAP1LC3B* (cor = 0.989, *p* < 0.001), *PINK1* (cor = 0.942, *p* < 0.001), *TOMM20* (cor = 0.744, *p* = 0.014), *PARK2* (cor = 0.858, *p* = 0.001), *TOMM22* (cor = 0.875, *p* = 0.001), *TOMM70A* (cor = 0.846, *p* = 0.002), *VDAC1*(cor = 0.750, *p* = 0.013), *CSNK2B* (cor = 0.805, *p* = 0.005), *MFN2* (cor = 0.948, *p* < 0.001), and *TOMM40* (cor = 0.772, *p* = 0.009). Additionally, the expression level of *COQ9* was significantly negatively correlated with the expression level of *SRC* (cor = -0.733, *p* = 0.016). Therefore, *COQ9* was selected for further analysis.

**Table 3:** The correlation between *ZNF764* and *COQ9* and mitochondrial autophagy-related genes expression.

Gene (s)	*ZNF764*		*COQ9*	
	ccorrelation coefficient	*p*	ccorrelation coefficient	*p*
*ATG12*	-0.361	0.305	0.195	0.588
*FUNDC1*	0.436	0.208	-0.136	0.707
*SQSTM1*	-0.333	0.347	0.955	< 0.001
*TOMM5*	-0.28	0.433	0.903	< 0.001
*UBB*	-0.168	0.642	0.772	0.009
*ATG5*	-0.629	0.051	0.744	0.014
*MAP1LC3A*	-0.39	0.265	0.914	< 0.001
*PGAM5*	-0.217	0.547	0.810	0.005
*SRC*	0.545	0.104	-0.733	0.016
*UBC*	-0.497	0.143	0.804	0.005
*MAP1LC3B*	-0.465	0.175	0.989	< 0.001
*PINK1*	-0.264	0.461	0.942	< 0.001
*TOMM20*	-0.087	0.811	0.744	0.014
*ULK1*	0.541	0.106	0.033	0.928
*CSNK2A2*	-0.7920	0.006	0.583	0.077
*MFN1*	-0.789	0.007	0.211	0.558
*PARK2*	-0.775	0.009	0.858	0.001
*TOMM22*	-0.508	0.134	0.875	0.001
*TOMM70A*	-0.458	0.183	0.846	0.002
*VDAC1*	-0.625	0.053	0.750	0.013
*CSNK2B*	-0.561	0.092	0.805	0.005
*MFN2*	-0.546	0.102	0.948	< 0.001
*RPS27A*	0.488	0.152	-0.029	0.936
*TOMM40*	-0.003	0.993	0.772	0.009
*UBA52*	-0.567	0.087	0.349	0.323

*p* < 0.05 was considered statistically significant.

### ceRNA regulatory network construction and verification

**Figure 6:**
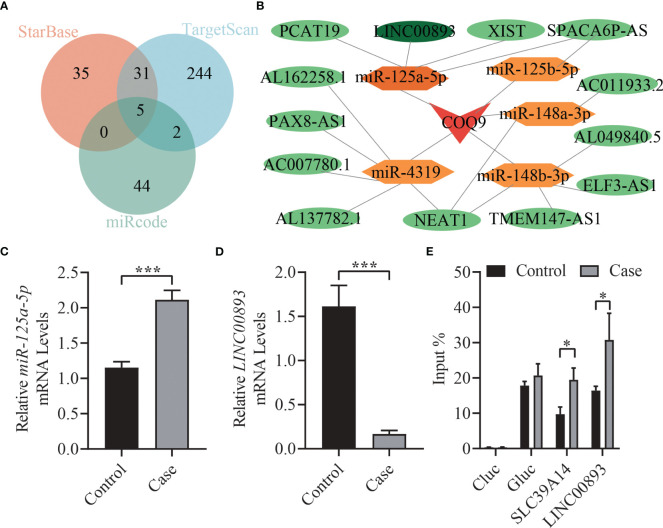
ceRNA regulatory network construction and verification. **(A)** Venn diagrams. Overlap of miRNAs in miRcode, TargetScan, and starBase databases. **(B)** ceRNA regulatory network. **(C)** The expression levels of miR-125a-5p in seminal exosomes of asthenozoospermic and normozoospermic groups via qRT-PCR. **(D)** The expression levels of *LINC00893* in seminal exosomes of asthenozoospermic and normozoospermic groups via qRT-PCR. **(E)** The m6A methylation level of *LINC00893* in seminal exosomes of asthenozoospermic and normozoospermic groups via qRT-PCR. ceRNA: Competitive endogenous RNA; qRT-PCR, quantitative reverse-transcription polymerase chain reaction. *p < 0.05; ***p < 0.001.

We utilized miRcode, TargetScan, and starBase databases to predict miRNAs that are potentially associated with *COQ9* and took intersections, including miR-125a-5p, miR-125b-5p, miR-148a-3p, miR-148b-3p, and miR-4139 (**Figure 6A**). Furthermore, lncRNAs potentially associated with these miRNAs were predicted using the starBase database, and a ceRNA regulatory network was constructed (**Figure 6B**). The expression levels of miR-125a-5p and *LINC00893* in exosomes of asthenozoospermic and normozoospermic individuals were detected, revealing higher expression of miR-125a-5p in asthenozoospermia and significantly decreased expression of *LINC00893* (*p* < 0.001, **Figures 6C, D**). Additionally, the expression of m6A methylation level of *LINC00893* was found to be significantly increased in patients with asthenozoospermia (*p* < 0.05, **Figure 6E**).

## Discussion

To explore potential biomarkers and pathways linked with asthenozoospermia, we performed bioinformatics analysis on GEO datasets and corroborated our observations through *in vitro* experiments. Our findings indicated that the expression of the *COQ9* gene was significantly lower in asthenozoospermia samples, and was associated with mitochondrial autophagy related genes expression. We constructed a ceRNA network consisting of *LINC00893*, miR-125a-5p, and *COQ9*. Furthermore, the vitro experiments demonstrated that *COQ9* and *LINC00893* exhibited low expression in asthenozoospermia, while miR-125a-5p showed high expression. Additionally, the m6A methylation level of *LINC00893* was found to be high in asthenozoospermia. These findings provided valuable insights into potential mechanisms underlying of asthenozoospermia.

Coenzyme Q9 (COQ9) is a precursor of coenzyme Q (CoQ) and plays a crucial role in maintaining mitochondrial function and cellular energy metabolism (18). Previous studies have shown that coenzyme Q10 (CoQ10), also known as ubiquinone, has important metabolic and antioxidant functions (19). Additionally, the concentration of CoQ10 in seminal plasma has been found to be directly correlated with sperm count and motility (20). Administering exogenous CoQ10 has been shown to improve sperm kinetic features in patients with idiopathic asthenozoospermia (21–23). Moreover, CoQ deficiency has been found to trigger the degradation of mitochondria through mitochondrial autophagy (24), while asthenozoospermia is primarily linked to mitochondrial dysfunction in sperm (3). Autophagy is closely associated with male reproduction, especially the biosynthetic and catabolic processes of sperm (25). By analyzing GEO datasets and experimental data, it was observed that the expression of *COQ9* was significantly lower in the asthenozoospermia group compared to the normal group. Furthermore, there was a significant positive correlation between *COQ9* expression and 16 mitochondrial autophagy-related genes expression. Based on these findings, it is speculated that the *COQ9* gene may affect COQ enzyme levels or mitochondrial autophagy, leading to mitochondrial dysfunction and impaired sperm motility, ultimately resulting in asthenozoospermia. However, further research is needed to fully understand the specific mechanism of *COQ9* in asthenozoospermia.

Furthermore, we conducted miRNA prediction to identify potential associations with *COQ9* and subsequently identified lncRNAs that are associated with these miRNAs. Finally, we constructed a ceRNA network. The overlapping miRNAs, including miR-125a-5p, miR-125b-5p, miR-148a-3p, miR-148b-3p, and miR-4139, were identified as *COQ9*-related miRNAs. Previous studies have found that miR-125a-5p is upregulated in the sperm of aging males, leading to increased cellular DNA damage in GC2 cells and perturbed stage-specific embryo development via Rbm38-p53 signaling (26). Sertoli cells provide protection and nutrition for developing sperm. miR-125a-5p regulated Sertoli cell proliferation and apoptosis by targeting *RAB3D* and regulating the PI3K/AKT pathway (27). Previous research reports only found that miR-148b-3p was downregulated in both human and mouse frozen-thawed sperm and was also decreased in embryos after fertilization using cryopreserved sperm (28). The association between miR-148a-3p, miR-4139, and sperm motility has not been reported. Therefore, we selected miR-125a-5p for further experimental verification and found that it is highly expressed in asthenozoospermia.

Additionally, four lncRNAs, including *XIST*, *PCAT19*, *SPACA6P-AS*, and *LINC00893*, were identified as miR-125a-5p-related lncRNAs in the ceRNA networks. XIST is a widely reported lncRNA that regulates X chromosome inactivation (XCI) and participates in the development of multiple types of tumors (29). *PCAT19* has also been associated with the development of multiple types of tumors (30–32). However, there are few reports about *SPACA6P-AS*, which has been identified as a prognostic biomarker of breast cancer through bioinformatics analysis (33), and has been found to interact with miR-125a in hepatocellular carcinoma (HCC) cells (34). *LINC00893* has previously been reported to be implicated in various malignancies. For instance, *LINC00893* o overexpression suppresses the proliferation, migration, or invasion of gastric cancer cells and regulates epithelial-mesenchymal transition by binding with *RBFOX2* (35). *LINC00893* inhibits the progression of prostate cancer through the miR-3173–5p/*SOCS3*/*JAK2*/*STAT3* pathway (36). *LINC00893* overexpression abrogates the proliferation and migration abilities of papillary thyroid cancer cells via the PTEN/AKT pathway (37). Moreover, *LINC00893* has been identified as an immune biomarker of sepsis through bioinformatic analysis (38). Our previous study, as well as the current study, has identified significantly lower expression of *LINC00893* compared to the control group. m6A methylation affects both mRNA and lncRNA, exerting diverse functions within cells. In the testes, m6A modification is involved in the regulation of spermatogenesis and development. Previous studies have demonstrated the significance of m6A modification in sperm formation, and aberrant m6A methylation has been associated with spermatogenesis disorders and infertility (39). In our study, we observed a significant upregulation of m6A methylation level of *LINC00893* in patients with asthenozoospermia. This finding suggests that m6A modification may play a pivotal role in the context of asthenozoospermia. Specifically, m6A methylation could potentially influence the stability and functionality of lncRNA, thereby impacting spermatogenesis and sperm motility. Nevertheless, the precise mechanism by which m6A methylation influences spermatogenesis and contributes to asthenozoospermia remains elusive, necessitating further investigations to unravel its underlying mechanisms.

However, it is important to acknowledge the limitations of our study. While we were able to verify the expression of *LINC00893*, miR-125a-5p, and *COQ9* in asthenozoospermia, we did not confirm their interaction relationship. Additional experiments should be conducted to validate the regulatory relationships among these molecules. Further studies *in vitro* and *in vivo* models are needed to elucidate the specific mechanism of the *LINC00893*-miR-125a-5p-*COQ9* ceRNA network in asthenozoospermia.

## Conclusions

In summary, this study identified *COQ9* as a marker gene in asthenozoospermia, associated with autophagy, ATP-dependent chromatin remodeling, endocytosis, and cell cycle. The ceRNA regulatory network (LINC00893/miR-125a-5p/COQ9) was constructed, revealing downregulation of *LINC00893* and *COQ9*, and upregulation of *miR-125a-5p* and m6A methylation level of *LINC00893* in asthenozoospermia compared to normozoospermic individuals. These findings provide valuable insights into the molecular mechanisms underlying asthenozoospermia.

## Data availability statement

The raw data supporting the conclusions of this article will be made available by the authors, without undue reservation.

## Ethics statement

The studies involving humans were approved by the Ethical Committee of the Hainan Women and Children’s Medical Center (No. 2023-134) and adhered to ethical standards set forth by the committee and the Declaration of Helsinki. All participants provided written informed consent after being fully informed of the study’s purpose. The studies were conducted in accordance with the local legislation and institutional requirements. Written informed consent for participation in this study was provided by the participants’ legal guardians/next of kin. Written informed consent was obtained from the individual(s), and minor(s)’ legal guardian/next of kin, for the publication of any potentially identifiable images or data included in this article.

## Author contributions

HL: Conceptualization, Investigation, Project administration, Writing – original draft. LZ: Formal analysis, Writing – original draft. AW: Formal analysis, Visualization, Writing – review & editing. HR: Methodology, Writing – review & editing. XC: Methodology, Writing – review & editing. YL: Resources, Writing – review & editing. JH: Resources, Writing – review & editing. WL: Conceptualization, Investigation, Writing – review & editing. MX: Conceptualization, Investigation, Writing – review & editing.

## Supplementary material

Supplementary Figure 1 Principal component analysis plot of the data for the different datasets.

Supplementary Figure 2 The Characterization of seminal plasma exosomes. **(A)** Transmission electron microscope image of seminal plasma exosomes. **(B)** The size distribution of seminal plasma exosomes. **(C)** The concentration of seminal plasma exosomes.

Supplementary Figure 3 Heatmap of the correlation between *ZNF764* and *COQ9* and autophagy-related genes expression.
