# Supplementary figures and images for: Identification of potential biomarkers and pathways for asthenozoospermia by bioinformatics analysis and experiments
Source: Front Endocrinol (Lausanne). 2024 May 28;15:1373774. doi: 10.3389/fendo.2024.1373774 (PMC11165088; doi:10.3389/fendo.2024.1373774)

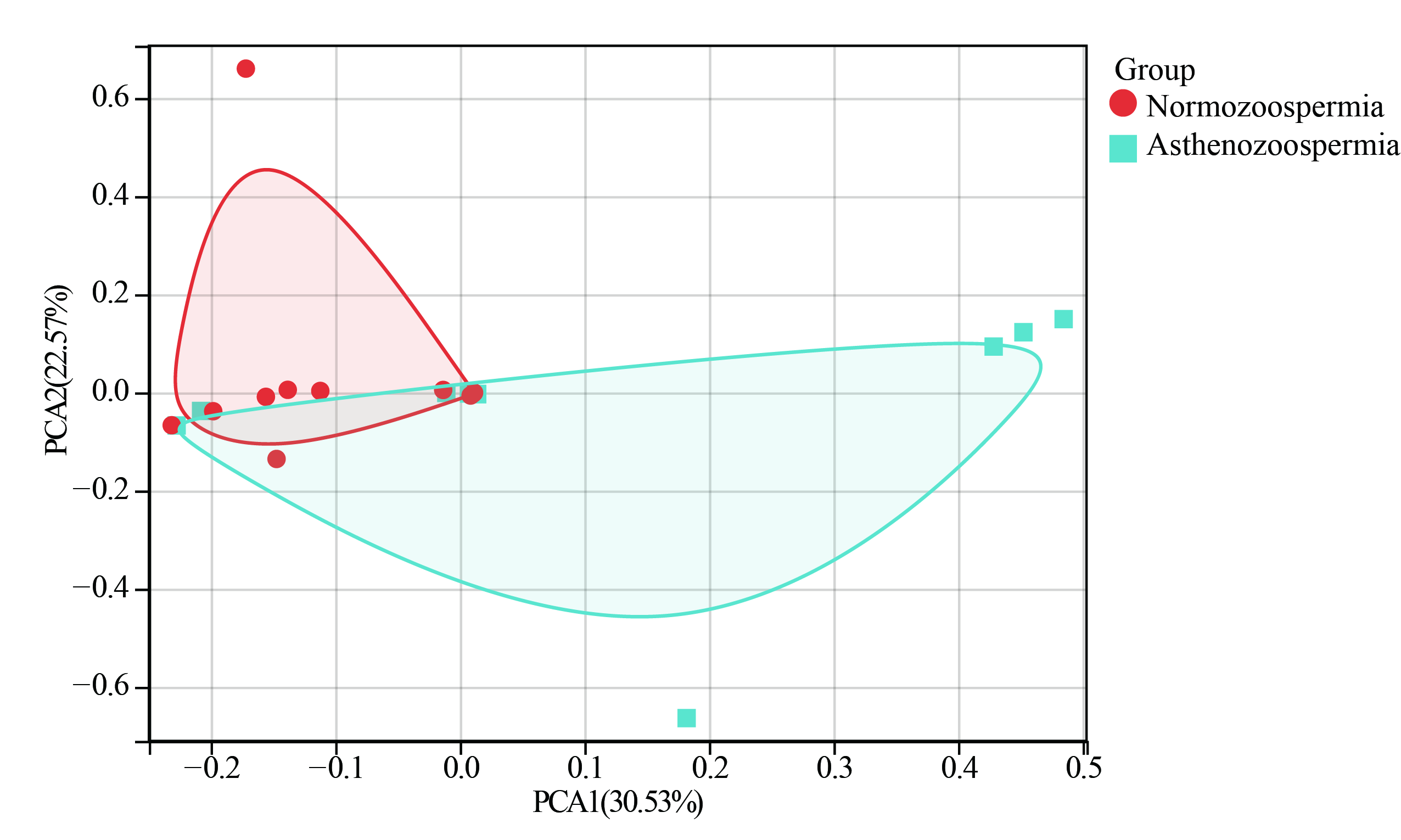

Supplement: Supplementary Figure 1 — Principal component analysis plot of the data for the different datasets. [file Image_1.tif]

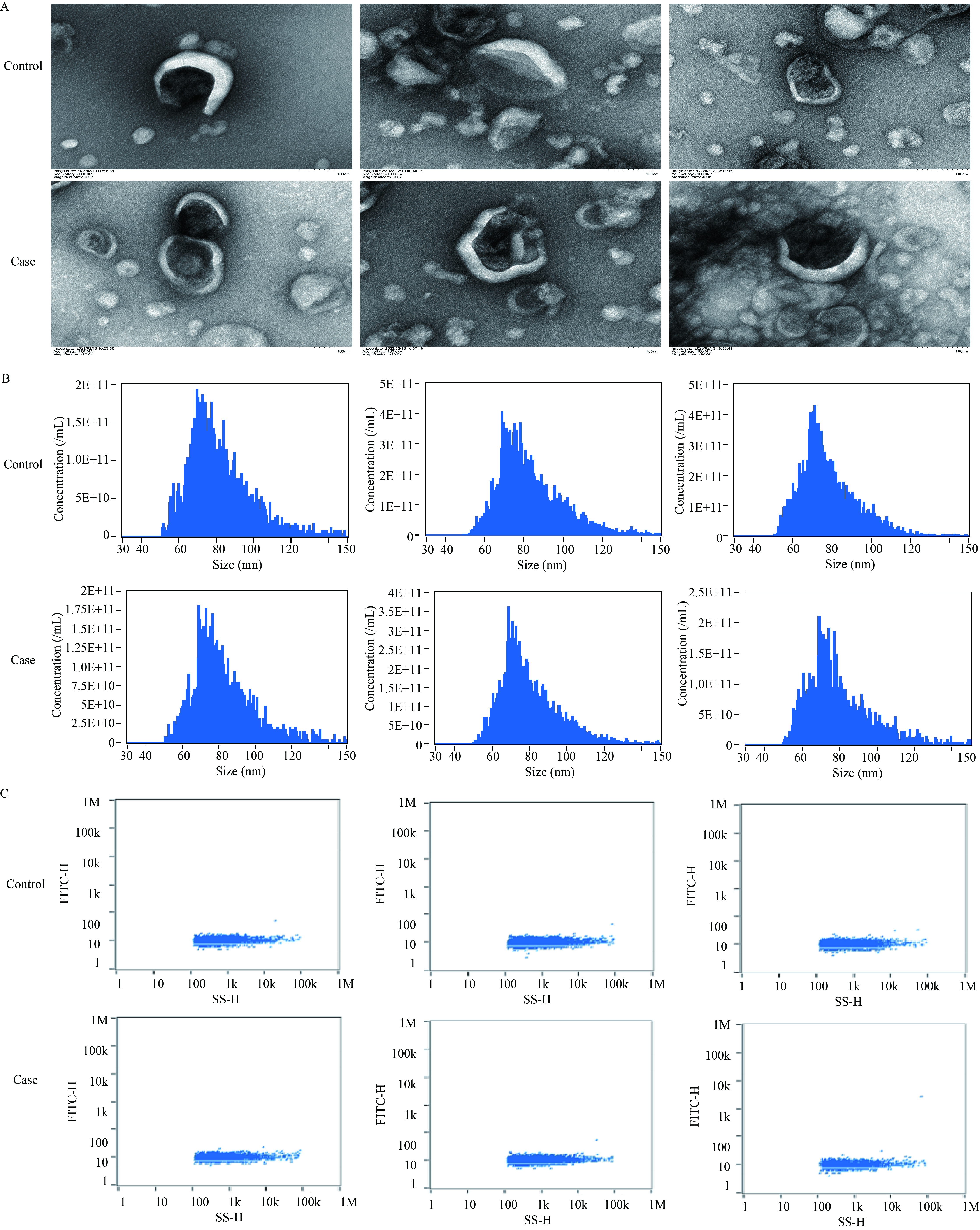

Supplement: Supplementary Figure 2 — The Characterization of seminal plasma exosomes. (A) Transmission electron microscope image of seminal plasma exosomes. (B) The size distribution of seminal plasma exosomes. (C) The concentration of seminal plasma exosomes. [file Image_2.tif]

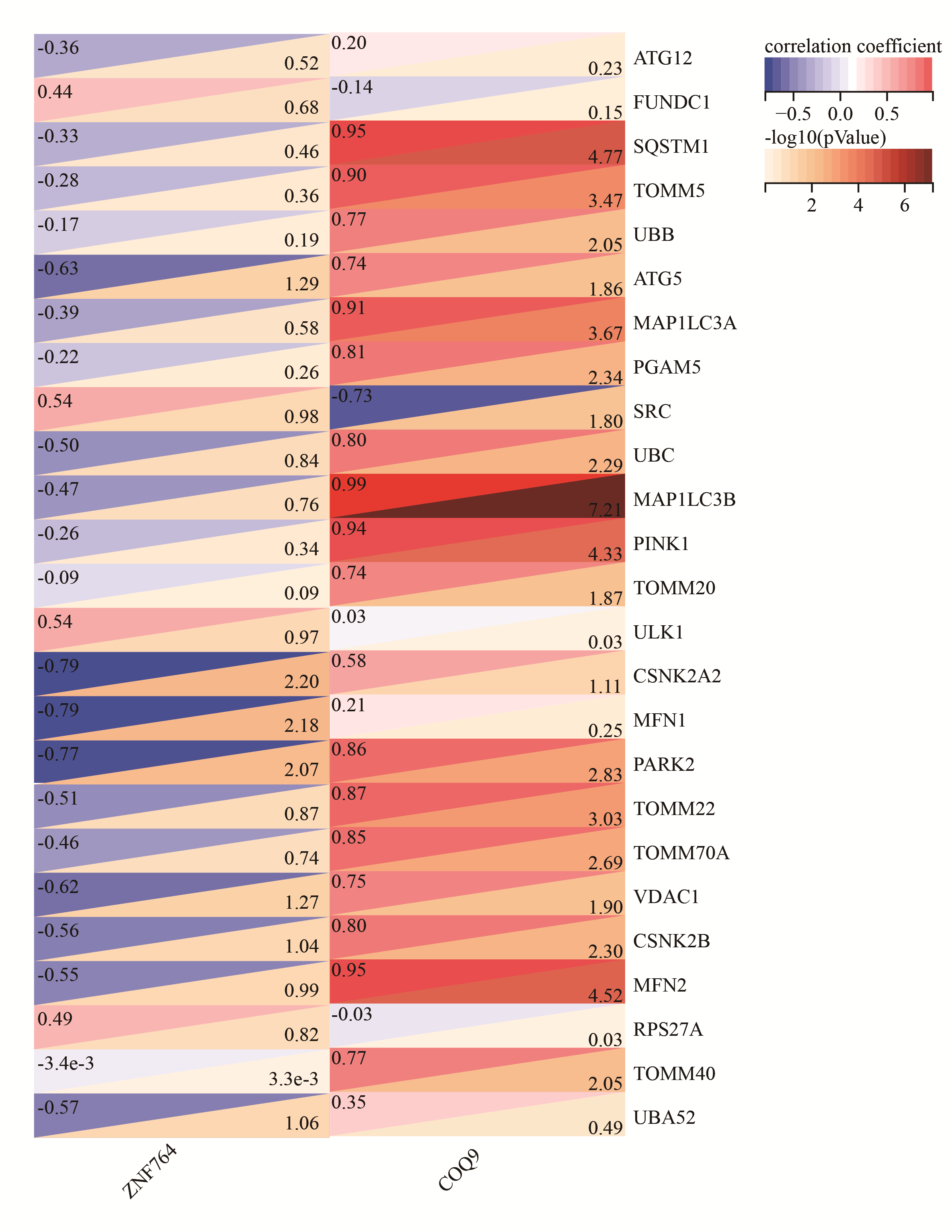

Supplement: Supplementary Figure 3 — Heatmap of the correlation between ZNF764 and COQ9 and autophagy-related genes expression. [file Image_3.tif]
